# The efficacy of acupuncture on suicidal behavior: A protocol for systematic review and meta-analysis

**DOI:** 10.3934/publichealth.2022046

**Published:** 2022-09-23

**Authors:** Chan-Young Kwon, Boram Lee

**Affiliations:** 1 Department of Oriental Neuropsychiatry, Dong-eui University College of Korean Medicine, 52–57, Yangjeong-ro, Busanjin-gu, Busan, Republic of Korea; 2 KM Science Research Division, Korea Institute of Oriental Medicine, 1672, Yuseong-daero, Yuseong-gu, Daejeon, Republic of Korea

**Keywords:** suicide, self-harm, acupuncture, acupressure, non-pharmacological treatment

## Abstract

**Background:**

One of the leading causes of death worldwide is suicide. Acupuncture has been reported to be related to clinical improvement of some risk factors for suicide including depression. Moreover, practitioner–patient communication is an important component of the acupuncture procedure, which may contribute to suicide risk reduction as a social contact. This systematic review was performed to evaluate the effectiveness and safety of acupuncture for suicidal behavior.

**Methods:**

A comprehensive search will be conducted in electronic medical databases including MEDLINE, the Cochrane Central Register of Controlled Trials, EMBASE, Allied and Complementary Medicine Database, PsycARTICLES, Cumulative Index to Nursing and Allied Health Literature, China National Knowledge Infrastructure, Wanfang data, VIP Chinese Science and Technology Periodicals, Citation Information by NII, Koreanstudies Information Service System, Korea Citation Index, Research Information Sharing Service, Oriental Medicine Advanced Searching Integrated System, and Korean Medical database. Interventional studies regardless of its design to assess the role of acupuncture on suicide prevention will be included. The validated measure of suicidal ideation including Beck scale for suicidal ideation will be considered as a primary outcome. The validated tools will be used to assess methodological quality of included studies according to its design (e.g., Cochrane Collaboration's risk of bias tool-2). If sufficient homogeneous data from controlled clinical trials exist, a quantitative synthesis will be performed. According to the heterogeneity of included studies, either a random-effects or fixed-effects model will be used.

**Discussion:**

The findings of this systematic review and meta-analysis will help to address the emerging major public health problem, suicide, in terms of evidence-based medicine.

## 1. Introduction

One of the leading causes of death worldwide is suicide, and suicide is considered an emerging public health problem. According to the World Health Organization World Mental Health Surveys, the twelve-month prevalence estimates of suicide ideation for developed countries and developing countries were 2.0% and 2.1%, respectively [1]. Typically, individual's mental disorders are known to be an important risk factor for suicidal behavior [2]. While mood disorders were the strongest predictors of suicide attempts in developed countries, substance abuse, post-traumatic stress disorder, and impulse control were the strongest predictors in developing countries [3]. Moreover, major physical health conditions including traumatic brain injury, sleep disorders, and back pain were also known to be associated with suicidal behavior [4]. On the other hand, socioecological models have pointed out social conditions such as inequities, discrimination, oppression, and historical trauma as risk factors for suicide [5].

As strategies for suicide prevention, there is clinical evidence that cognitive behavioral therapy and dialectical behavior therapy for suicide prevention have modest benefit in reducing suicidal ideation and suicide attempts [6]. As pharmacotherapies for suicide prevention, although there is some evidence that lithium and clozapine are helpful in preventing suicide in some psychiatric conditions, the role of mood stabilizers, sedatives or hypnotics, and antidepressants in preventing suicidal behavior is controversial [7]. Moreover, some social strategies including restricting access to lethal means and avoiding the Werther effect (imitation suicide) are also considered to be important strategies of suicide prevention [8]. Therefore, suicide prevention requires a multifaceted strategy at the national level, not just in the clinic setting [9].

However, about three-fifths of individuals with suicide behavior (i.e., suicide ideation, plans and attempts) do not receive appropriate treatment, the most common reasons being low perceived need and the wish to handle the problem alone [10]. In this context, suicide attempters have an overwhelmingly higher percentage of primary care visits than mental health services prior to suicide completion [11]. This suggests that the role of non-psychiatric clinics including primary care center in appropriately assessing and managing patients' suicide risk and referring them to specialized mental health services when necessary are important in suicide prevention strategies at the national level [11].

**Figure 1.**
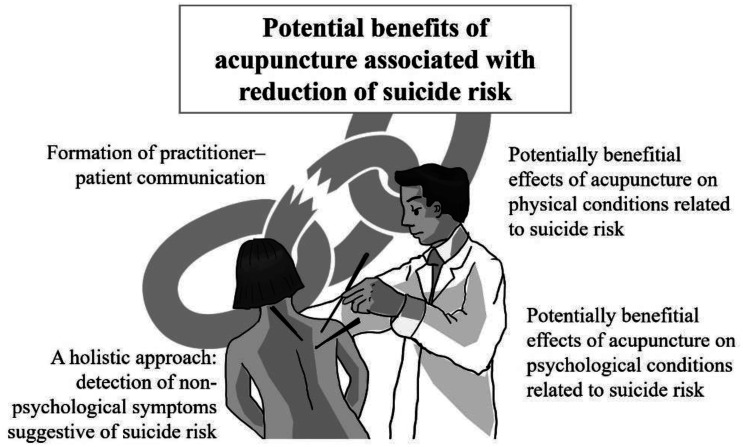
Potential benefits of acupuncture for suicide prevention.

Considering that the prevalence of complementary and alternative medicine (CAM) use is also high at 35% among individuals with mental health problems, it has important public health implications to appropriately screen and manage high-risk groups for suicide in facilities or clinics using CAM modalities [12]. Acupuncture is a representative type of CAM and has been reported to be related to clinical improvement of some risk factors for suicide such as chronic pain [13], depression [14], and post-traumatic stress disorder [15]. Moreover, practitioner–patient communication is an important component of the acupuncture procedure [16], which may contribute to suicide risk reduction as a social contact (Figure 1). Given the potential benefits of CAM modalities including acupuncture in suicide prevention, it is urgent to explore the applicability of this promising strategy from the perspective of evidence-based medicine. That is, to be considered a suicide prevention strategy, there is a prerequisite that it should conform to an evidence-based approach [9]. In this context, there is a recent systematic review that found that mindfulness-based intervention, a CAM modality, may contribute to the reduction of suicidal behavior [17]. However, there are no studies that comprehensively investigated the role of acupuncture on suicide prevention.

This systematic review will be performed to investigate the effectiveness and safety of acupuncture for suicidal behavior. This study may contribute to establishing a suicide prevention strategy using this non-pharmacological treatment for suicide prevention in countries that use acupuncture as a mainstream medical system or as a CAM modality.

## 2. Materials and methods

### 2.1. Protocol

The protocol of this systematic review is registered in the Open Science Framework (registration number, 3EKN9) as well as PROSPERO (registration number, CRD42022334375). When protocol amendments occur, dates, changes, and rationales of the amendments will be tracked in the registry. This protocol followed the Preferred Reporting Items for Systematic Review and Meta-Analysis Protocols 2015 statement [18].

### 2.2. Data sources and search strategy

One researcher (CY Kwon) will search the following 15 electronic bibliographic databases from their inception dates to July 2022: MEDLINE (via PubMed), the Cochrane Central Register of Controlled Trials, EMBASE (via Elsevier), Allied and Complementary Medicine Database (via EBSCO), PsycARTICLES (via ProQuest), Cumulative Index to Nursing and Allied Health Literature (via EBSCO), China National Knowledge Infrastructure, Wanfang data, VIP Chinese Science and Technology Periodicals, Citation Information by NII, Koreanstudies Information Service System, Korea Citation Index, Research Information Sharing Service, Oriental Medicine Advanced Searching Integrated System, and Korean Medical database. Also, to find potentially missing literature, the authors will review the reference lists of the relevant review articles as well as included studies, and will perform manual search on Google Scholar. The literature search in this systematic review will be comprehensive and tailored to each database and each language. The search strategy in MEDLINE is in Table 1.

**Table 1.** Search strategies for the Medline via PubMed.

Number	Content
#1	Suicide [MeSH] OR “Self Mutilation” [MeSH] OR self-harm OR self-poisoning OR self-injur* OR “Self Mutilation” OR “attempted suicide” OR suicid*
#2	Acupuncture[MeSH] OR Acupressure[MeSH] OR “Acupuncture Therapy”[MeSH] OR “Acupuncture Points”[MeSH] OR acupunct* OR electroacupunct* or electro-acupunct* OR acupoint* OR acupressure OR auriculotherapy
#3	#1 AND #2

### 2.3. Eligibility criteria

The following inclusion criteria will be used in this systematic review. (1) Types of studies: Given that it is difficult to conduct randomized controlled clinical trials using suicidal behavior as an outcome, and meaningful results can be found not only in randomized controlled clinical trials but also in other intervention studies besides randomized controlled clinical trial, this systematic review will allow original interventional studies regardless of its study type. Therefore, randomized or non-randomized controlled clinical trials (RCTs or CCTs) and before-after studies will be allowed. However, retrospective observational studies such as case reports/case series will not be allowed. There will be no restrictions on the publication language and publication status of documents. In other words, gray literature such as conference abstracts and dissertations will be allowed. (2) Types of participants: There are no restriction on the types of participants. That is, both the clinical population and the non-clinical population will be allowed. Moreover, there will be no restrictions on the gender/sex, age, or ethnicity/race of the participants. (3) Types of interventions: Any type of acupuncture will be allowed. That is, studies involving manual acupuncture, ear acupuncture, scalp acupuncture, electroacupuncture and acupressure with or without routine care will be allowed. In addition to acupuncture as monotherapy, the use of acupuncture as part of multicomponent interventions (i.e., as adjunctive therapy) will be acceptable. However, acupoint injection or pharmacopuncture will not be considered for inclusion in acupuncture in this study. That is, studies using only acupoint injection or pharmacopuncture will be excluded. (4) Types of controls: For studies with controls, no treatment, wait-list, sham control (i.e., sham acupuncture), and active comparators (e.g., antidepressants) will be allowed as their control group. (5) Types of outcome: Primary outcome will include any validated measure of suicidal ideation including Beck scale for suicidal ideation [19]. Secondary outcomes will include any other measures of suicidal behaviors including suicidal ideation, attempts, or complete.

This content of this study can be summarized in the form of Population-Intervention-Comparison-Outcome (PICO) [20]. The PICO of this systematic review is as follow. Population: no restriction; Intervention: acupuncture; Comparison: active controls, sham treatment, wait-list and no treatment; Outcome: suicidal behaviors including suicidal ideation, attempts, or complete.

### 2.4. Study selection process

**Figure 2.**
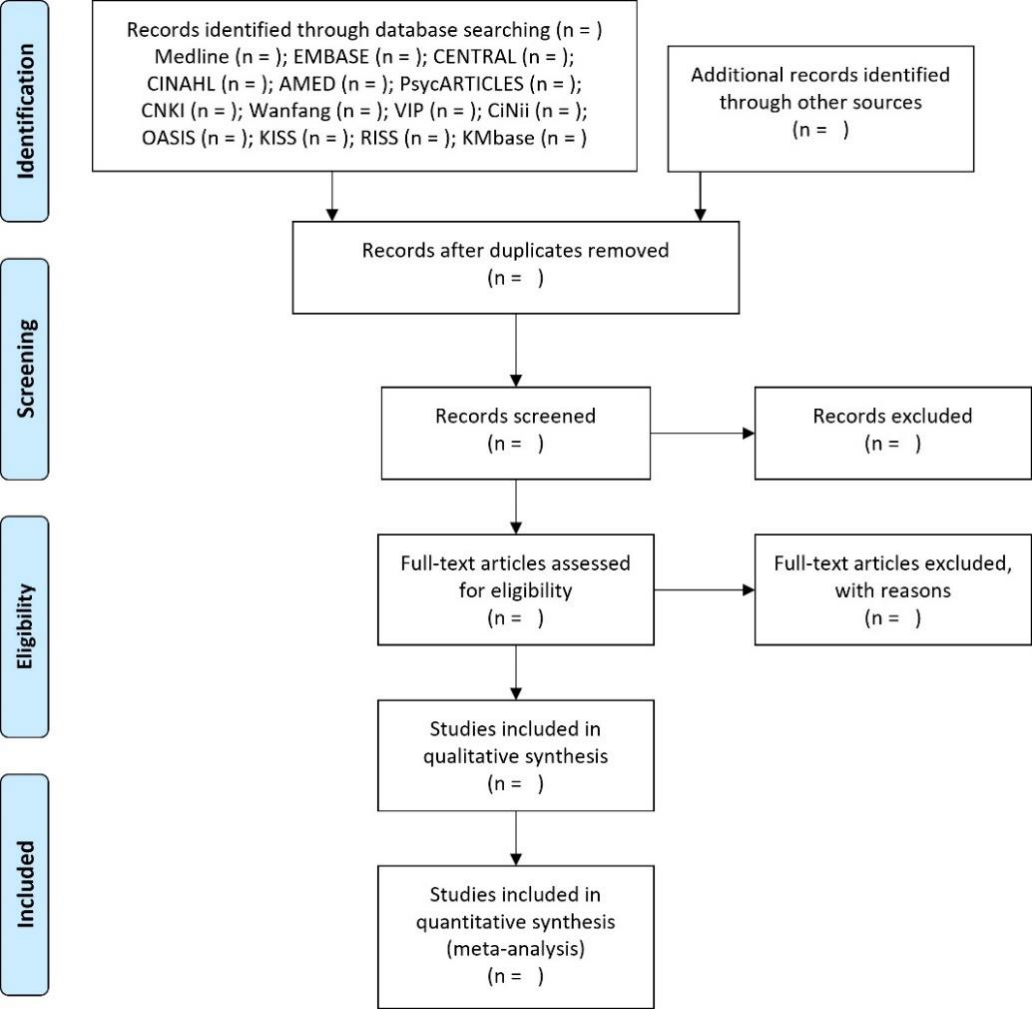
A PRISMA flow diagram of the literature screening and selection processes. *Note: AMED: Allied and Complementary Medicine Database; CENTRAL: Cochrane Central Register of Controlled Trials; CINAHL: Cumulative Index to Nursing and Allied Health Literature; CNKI: China National Knowledge Infrastructure; KISS: Koreanstudies Information Service System; OASIS: Oriental Medicine Advanced Searching Integrated System; RISS: Research Information Service System.

In the study selection process, there will be a two-step process. In the first step, two independent researchers (CY Kwon and B Lee) will screen the titles and/or abstracts of searched documents to identify potentially relevant studies. After the initial screening, the same two researchers will independently review the full texts of the screened studies for eligibility. Any disagreements in the process will be resolved through their discussion. If the disagreements are not resolved through their discussion, a third reviewer (HW Suh) will intervene. EndNote20 (Clarivate Analytics, Philadelphia, USA) will be used to manage quotations. The PRISMA flow diagram will be used to visualize the study selection process (Figure 2).

### 2.5. Data extraction process

Two independent researchers (CY Kwon and B Lee) will perform the data extraction process, by using a standardised, pre-defined and pilot-tested Microsoft Excel (Microsoft, Redmond, WA, USA) form. Following information will be extracted from the included studies: sample size, characteristics of participants, types of experimental and control interventions, outcome measures and the results, adverse events or safety profile, and information for assessment of methodological quality of the study. Any discrepancies between the researchers will be identified and resolved through their discussion. If an agreement is not reached through their discussion, a third reviewer (HW Suh) will intervene. We will contact the first and/or corresponding authors of the studies via e-mail, when there are missing or unclear data.

### 2.6. Quality assessment process

Depending on the type of studies included, corresponding risk of bias assessment tools will be used to assess its methodological quality. For example, the revised risk of bias tool [21] developed by the Cochrane group will be used to assess the quality of RCTs. In this tool, five distinct domains including bias due to randomization process, intended interventions, missing outcome data, measurement of the outcome, and selection of the reported result, will be judged to “low risk of bias,” “some concerns,” or “high risk of bias” [21]. The Risk of Bias Assessment tool for Non-randomized Study tool will be used to assess the quality of CCTs [22]. In this tool, eight distinct domains including the possibility of the target group comparisons, target group selection, confounder, exposure measurement, blinding of assessors, outcome assessment, incomplete outcome data, and selective outcome reporting will be judged to “low”, “high”, or “unclear risk of bias” [22]. Finally, the Quality Assessment Tool for Before-After (Pre-Post) Studies With No Control Group will be used to assess the quality of before-after studies [23]. In this tool, the quality of included before-after study will be judged as “good”, “fair”, or “poor”, by evaluating 12 defined criteria [23]. Two independent researchers (CY Kwon and B Lee) will perform the quality assessment process, and any discrepancies will be resolved through their discussion. If the discrepancies are not resolved through their discussion, a third reviewer (HW Suh) will intervene.

### 2.7. Data synthesis and analysis process

Qualitative analysis will be performed for all included studies. When sufficient homogeneous data from controlled clinical trials including RCT and CCT exist, a quantitative synthesis will be performed. A meta-analysis will be conducted using the Cochrane group's RevMan 5.4 (the Cochrane Collaboration, London, UK). Dichotomous and continuous data will be presented as a risk ratio with 95% confidence intervals (CIs) and mean differences with 95% Cis, respectively. By using both the χ^2^ test and the I^2^ statistic, statistical heterogeneity of effect measures from meta-analysis will be identified. The I^2^ values of ≥ 50% will be considered as substantial statistical heterogeneity, while I^2^ values of ≥ 75% will be considered as considerable statistical heterogeneity. The method of meta-analysis will be determined taking into account the calculated statistical heterogeneity and clinical heterogeneity between included studies. That is, a random-effect model will be used if the results have substantial statistical heterogeneity (I^2^ value ≥ 50%), or if sufficient clinical homogeneity of studies included in meta-analysis is not guaranteed. Moreover, if the number of studies included in the meta-analysis is small (n < 5), or if the calculated statistical heterogeneity is not substantial, the fixed-effect model will be used [24],[25].

To address potential heterogeneity of included studies, subgroup analyses are planned according to following criteria: type of population (clinical or non-clinical), and treatment duration. Moreover, to identify the robustness of the results of meta-analysis, sensitivity analyses are planned excluding studies with poor methodological quality or studies with numerically distant outliers from the rest of the data.

In addition, by conducting a cumulative meta-analysis, we will evaluate the sufficiency and stability of the meta-analysis dataset over time as studies are continuously added [26]. For conducting cumulative meta-analysis, the command “metacum” in Stata 14.0 software (StataCorp, TX, USA) will be used, studies will be added alphabetically by year of publication, and a random-effects model will be applied.

### 2.8. Assessment of reporting bias

If sufficient studies are available (n ≥ 10) in a meta-analysis, the evidence of publication bias will be investigated by generating funnel plot. Also, Egger's regression test will be used to quantitatively investigate the potential reporting bias, by using Stata 14.0 software (StataCorp, TX, USA). If publication bias is detected, the trim and fill method will be used to correct and adjust the publication bias [27].

### 2.9. Ethics approval of research

The present review protocol did not involve individual patients or public agencies.

## 3. Discussion

Efforts at the national level are needed to overcome the problem of suicide, the leading cause of death worldwide [1]. Given the high prevalence of CAM use and the high visitation rate of non-psychiatric clinics among suicide victims and high-risk groups for suicide [11],[12], it is important to identify the benefit-risk ratio of CAM modalities in reducing suicide risk from the perspective of evidence-based medicine. As a kind of CAM modalities, acupuncture has been reported to improve some physical and mental health known to be associated with suicide risk [13]–[15], so it can be considered as an potentially promising non-pharmacological modality for the establishment of future suicide prevention strategies. However, as far as we know, there has been no to investigate roles of acupuncture in reducing suicidal behavior. The findings of this systematic review and meta-analysis will help to address the emerging major public health problem, suicide, in terms of evidence-based medicine.

Currently, acupuncture has become a popular intervention not only in Asian countries such as China, Taiwan, Japan, and Korea, but also in Western countries including the United States [28]. Aside from the clinical effect of acupuncture, the potential strength of this non-pharmacological treatment in suicide prevention is that it requires frequent face-to-face contact by practitioner–patient [16]. In this contact, the practitioner generally conducts a comprehensive assessment including assessment of mental health from a holistic point of view as well as the patient's chief complaints [29]. Given the importance of mobilization of multidisciplinary personnel including primary care as well as psychiatric clinics for suicide prevention [11], elucidating the role of acupuncture in suicide prevention is potentially important to policy utilization of clinicians or practitioners using acupuncture in the national suicide prevention strategies.

However, in order for this CAM modality to be utilized in the national suicide prevention policy, it is necessary to verify it from the point of view of evidence-based medicine [9]. In that sense, the results of this systematic review are thought to be helpful for decision-making not only by clinicians but also by policy makers by analyzing the efficacy of acupuncture on suicidal behavior comprehensively and critically. The results of this systematic review and meta-analysis will be published in a peer-reviewed journal.
